# Pressure-Induced Magnetic Crossover Driven by Hydrogen Bonding in CuF_2_(H_2_O)_2_(3-chloropyridine)

**DOI:** 10.1038/srep06054

**Published:** 2014-08-13

**Authors:** Kenneth R. O'Neal, Tatiana V. Brinzari, Joshua B. Wright, Chunli Ma, Santanab Giri, John A. Schlueter, Qian Wang, Puru Jena, Zhenxian Liu, Janice L. Musfeldt

**Affiliations:** 1 Department of Chemistry, University of Tennessee, Knoxville, Tennessee 37996 USA; 2 Geophysical Laboratory, Carnegie Institution of Washington, Washington D.C. 20015 USA; 3 State Key Laboratory of Superhard Materials, Jilin University, Changchun 130012 China; 4 Physics Department, Virginia Commonwealth University, Richmond, Virginia 23284 USA; 5 Materials Science Division, Argonne National Laboratory, Lemont, Illinois 60439 USA; 6 Division of Materials Research, National Science Foundation, Arlington, Virginia 22230 USA; 7 Department of Physics, University of Florida, Gainesville, FL 32611-8440, USA; 8 Center for Applied Physics and Technology of Peking University, Beijing 100871, China

## Abstract

Hydrogen bonding plays a foundational role in the life, earth, and chemical sciences, with its richness and strength depending on the situation. In molecular materials, these interactions determine assembly mechanisms, control superconductivity, and even permit magnetic exchange. In spite of its long-standing importance, exquisite control of hydrogen bonding in molecule-based magnets has only been realized in limited form and remains as one of the major challenges. Here, we report the discovery that pressure can tune the dimensionality of hydrogen bonding networks in CuF_2_(H_2_O)_2_(3-chloropyridine) to induce magnetic switching. Specifically, we reveal how the development of 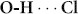 exchange pathways under compression combined with an enhanced *ab*-plane hydrogen bonding network yields a three dimensional superexchange web between copper centers that triggers a reversible magnetic crossover. Similar pressure- and strain-driven crossover mechanisms involving coordinated motion of hydrogen bond networks may play out in other quantum magnets.

The interplay between charge, structure, and magnetism leads to rich phase diagrams and highly tunable properties in multifunctional materials^1^,^2^. This is because exotic properties tend to emerge when phases compete. Molecule-based materials are particularly revealing in this regard due to their low energy scales and sensitivity to various external stimuli like temperature, pressure, and magnetic field^3^,^4^,^5^,^6^,^7^,^8^,^9^,^10^,^11^,^12^. Another characteristic of molecule-based materials is their tendency to develop hydrogen bonding networks^13^,^14^,^15^,^16^,^17^,^18^,^19^,^20^,^21^,^22^,^23^,^24^,^25^,^26^,^27^,^28^. Pressure and strain are especially attractive tuning parameters in this case because they act directly on bond lengths and angles as well as the hydrogen bonding pattern.

While magnetic exchange interactions are traditionally established through direct and superexchange mechanisms between metal centers or metal centers and various ligands^29^, exchange can also occur through intermolecular hydrogen bonding^14^,^15^,^16^,^17^,^18^,^19^,^20^,^21^,^22^,^23^,^24^,^25^,^26^,^27^,^28^. Physical examples include coordination polymers and low-dimensional systems like Cu(pyz)(NO_3_)_2_, Cu(pyz)F_2_(H_2_O)_2_, and CuX_2_(pyrazine-N,N′-dioxide) (H_2_O)_2_ (X = Cl, Br), all of which display transitions that involve hydrogen bonding networks. Changes in bond lengths and angles along the magnetic exchange pathway affect the hopping integrals between magnetic centers, thereby altering the magnetic exchange *J*^29^,^30^. One might suspect that pressure-driven local lattice distortions modify the hydrogen bonds and thus the magnetism, although direct evidence for this mechanism is rare. CuF_2_(H_2_O)_2_(3-chloropyridine) attracted our attention due to its pentacoordinate copper environment, two-dimensional hydrogen bonding network, and pressure-induced magnetic transition that allows us to test this supposition^31^ (Schlueter, J. A. Unpublished work). This system differs from the prior examples in that the structure is fully molecular rather than being covalently bound (Fig. 1(a)). Moreover, it displays a buckled network of intermolecular hydrogen bonds between the H_2_O ligands and fluoride centers that act as superexchange linkages between the copper centers within the *ab* plane. This network facilitates antiferromagnetic ordering below 2.1 K^31^. There is evidence that the system displays ferromagnetic behavior under pressure (0.8 GPa)(Schlueter, J. A. Unpublished work).

By combining diamond anvil cell techniques, high pressure infrared and Raman spectroscopies, and complimentary calculations of energy, local structure trends, and lattice dynamics, we uncover the ability of hydrogen bond formation to trigger the antiferromagnetic to ferromagnetic crossover in CuF_2_(H_2_O)_2_(3-chloropyridine). Our analysis reveals that compression enhances the in-plane 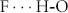 exchange and creates new intermolecular hydrogen bonds between chlorine on the pyridine ring and the hydrogen centers on the water ligands. The latter pathway forms because compression reduces interatomic distances, aligns the Cl-containing ring, and widens the H_2_O ligands, leading to a three dimensional hydrogen bonding network between copper centers. This increased superexchange network dimensionality drives the 0.8 GPa magnetic crossover. This process is reversible, meaning that when pressure is released, the extra exchange pathway is eliminated. We conclude that magnetic tunability in CuF_2_(H_2_O)_2_(3-chloropyridine) derives from and depends upon the presence of flexible intermolecular hydrogen bonding networks. Further compression reveals another distortion between 4 and 5.5 GPa involving the bipyramidal copper environment although, at this time, it is not known whether there is a magnetic component. In addition to establishing how pressure-induced changes in bond lengths and angles control magnetism in hydrogen bonded quantum magnets like CuF_2_(H_2_O)_2_(3-chloropyridine), these findings are important for unraveling spin crossover processes and energy transfer mechanisms in other functional materials like multiferroics.

## Results

Figure 2 (a,c) displays close-up views of the infrared and Raman spectra of CuF_2_(H_2_O)_2_(3-chloropyridine) between ambient pressure and 1.5 GPa. Both sets of spectra show signatures of the 0.8 GPa transition. With increasing pressure, the 125 cm^−1^ infrared active lattice mode diminishes and then disappears. The displacement pattern for this mode is highly collective and involves the F-Cu-F symmetric stretch, the O-Cu-O asymmetric bend, and libration of the 3-chloropyridine ring around the C-Cl bond. Turning to the Raman response, a shoulder around 1575 cm^−1^, which we assign as a combination of C = C and C-N in-phase, in-plane stretches and C-Cl rocking motion, also diminishes and then disappears. Figure 2 (b,d) shows frequency versus pressure plots for these structures. Their disappearance through the 0.8 GPa transition indicates that the lattice is sensitive to the magnetic crossover, a sign of magnetoelastic coupling^32^,^33^,^34^,^35^. Note that we employ room temperature, high pressure data to understand the low temperature response because the spectra are nearly insensitive to temperature. Although the magnetic crossover is observed at low temperatures, our variable temperature measurements show minimal spectral changes down to 4 K (see Supplemental Material), even through the orthorhombic (*Pmma*) to monoclinic (*P2*_1_/*c*) transition takes place between 200 and 100 K. This allows the use of room temperature, high pressure data to understand the low temperature magnetic crossover..

We carried out lattice dynamics calculations in order to assign the vibrational modes of CuF_2_(H_2_O)_2_(3-chloropyridine) and relaxations to model structural changes between the low-pressure antiferromagnetic and high-pressure ferromagnetic states (Fig. 3 (a–c)) (Schlueter, J. A. Unpublished work). Our calculations predict that the ferromagnetic state becomes energetically favorable above 0.75 GPa, in excellent agreement with the 0.8 GPa crossover found experimentally. As anticipated, most interatomic distances decrease under compression. The drastic decrease in the 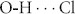 distance with pressure is particularly striking. A small discontinuity also appears at the critical pressure. Moreover, one O-H bond length is predicted to increase while the other decreases (such that they become more similar), and the H-O-H bond angle widens dramatically. Taken together, our simulations suggest that changes in the 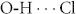 distance and shape of the H_2_O ligands are the most important local lattice distortions through the 0.8 GPa transition.

Figure 3 (d–f) displays frequency versus pressure trends for three different vibrational modes: the C-Cl stretches, the H-O-H bend, and the O-H stretches. As our calculations predict, these features are sensitive to the transition. For instance, on approach to the 0.8 GPa transition, the C-Cl stretching modes blue shift with increasing pressure. Above the critical pressure, these same vibrational modes display a much smaller d*ω*/dP, indicating the stabilization of a less compressible phase. At the same time, the H-O-H bend hardens significantly over the entire pressure range, consistent with the prediction of increasing angle^36^. There is also a notable change in slope through the transition regime. Finally, both O-H stretching features soften under pressure, although at different rates. Softening is characteristic of improved hydrogen bonding interactions^15^,^37^, and the divergent rates imply that the two O-H stretching modes in the water ligand are becoming more inequivalent. The latter trend is in apparent contradiction to the aforementioned prediction of one O-H bond lengthening and the other shortening. As discussed below, this observation has its origin in the breakdown of simple frequency-bond length correlations^34^,^38^,^39^.

**Figure 1:**
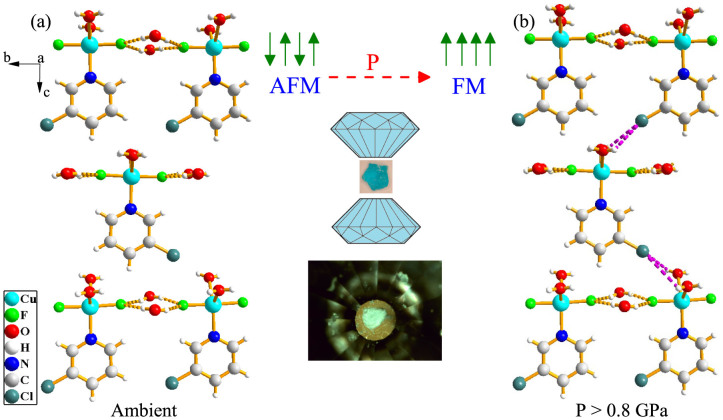
(a) Crystal structure of CuF_2_(H_2_O)_2_(3-chloropyridine) at 10 K showing the buckled two-dimensional hydrogen bonded layers^31^. Parts of neighboring CuF_2_(H_2_O)_2_(3-chloropyridine) molecules have been omitted to emphasize the hydrogen bonding. (b) Schematic rendering of the structure above 0.8 GPa illustrating the three dimensional network that is formed under compression. The connection in the third direction consists of intermolecular 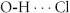 hydrogen bonds, as indicated by the purple dashed lines. Also included are drawings of the pressure-induced magnetic crossover and diamond anvil cell as well as a photo of CuF_2_(H_2_O)_2_(3-chloropyridine) on the diamond culet.

**Figure 2:**
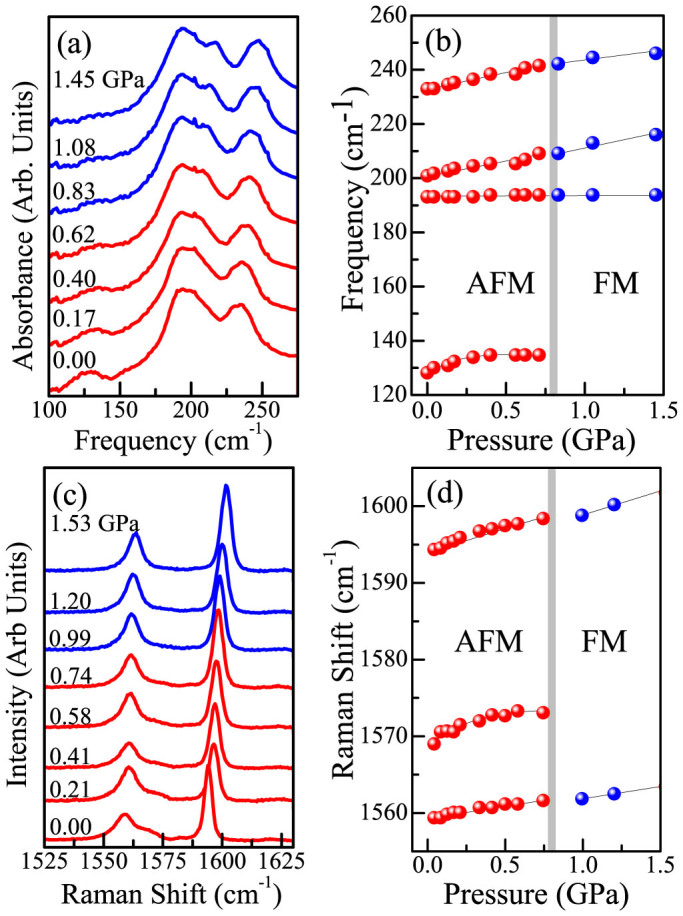
(a) Infrared spectra of CuF_2_(H_2_O)_2_(3-chloropyridine) at 300 K and various pressures demonstrating the disappearance of the 125 cm^−1^ lattice mode through the 0.8 GPa transition. (b) Frequency versus pressure for the infrared active modes in panel (a). (c) Room temperature Raman spectra at the indicated pressures showing the disappearance of a shoulder around 1565 cm^−1^. (d) Frequency versus pressure for the Raman active modes in panel (c). The vertical grey line marks the critical pressure for the 0.8 GPa magnetic crossover.

**Figure 3:**
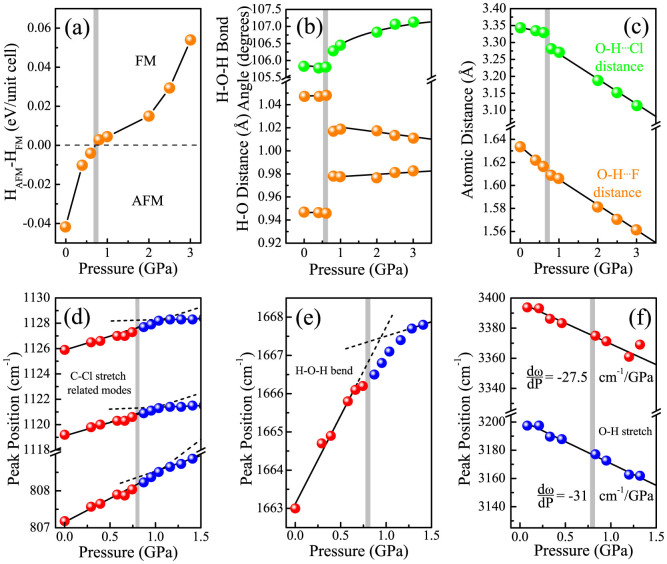
(a) Relative enthalpy of the CuF_2_(H_2_O)_2_(3-chloropyridine) unit cell, predicting that the ferromagnetic state becomes energetically favored. (b) Calculated H-O bond distances and H-O-H bond angle and (c) 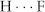 and 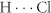 distances all indicate sharp changes at the critical pressure. The grey lines indicate the transition pressure which is in excellent agreement with the experimental pressure (0.75 vs. 0.8 GPa). (d) Experimental frequency versus pressure at 300 K for three modes involving the C-Cl bond, (e) H-O-H bend, and (f) O-H stretches. All modes involving the C-Cl bond show slight increases in d*ω*/dP around the transition. The H-O-H bend hardens significantly with pressure. The difference in d*ω*/dP (−27.5 ± 2 vs. −31 ± 1 cm^−1^/GPa) for the two O-H stretches results in increased splitting between the features. Lines are drawn to guide the eye and help visualize different mode behaviors through the transition.

Taken together, we find that hydrogen bonds are established between the H_2_O ligands and the Cl center through the 0.8 GPa magnetic transition (Fig. 1 (b)). The shortened 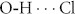 distance falls within the range of a “long” hydrogen bond with chlorine^40^, which explains the hardening of the C-Cl stretching modes as the motion is dampened by the new interaction and increased stability beyond 1 GPa. This interaction also accounts for the disappearance of the 125 cm^−1^ infrared mode seen in Figure 2 (a,b) since the 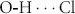 hydrogen bonds prevent the 3-chloropyridine ring from librating. Since the chlorine center is closer to one hydrogen than the other, the hydrogen bond forces the H-O-H angle to open, dampening the bending motion. This process hardens the H-O-H bending mode. What is formed in the end is essentially an asymmetric pair of O-H bonds (from the point of view of a single H_2_O ligand), in excellent agreement with our calculations (Fig. 3). The establishment of new hydrogen bonding also accounts for the prediction of one O-H bond lengthening and the other shortening (Fig. 3(b)). As the hydrogen closer to the chlorine is pulled away from the oxygen center, the bond length of the second O-H linkage ought to be reduced as the electrostatic repulsion is lessened. The intermolecular 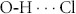 hydrogen bond also shifts the electron density of the oxygen towards chlorine, effectively reducing bond order between the oxygen and the hydrogen center that is not interacting with the chlorine. This is evidenced in our spectra by increased splitting between the two O-H stretching modes as pressure is applied (−27.5 ± 2 vs. −31 ± 1 cm^−1^/GPa).

We propose that intermolecular hydrogen bonding between the water ligand and chlorine acts as an additional superexchange pathway between copper centers along the *c* axis, adding a third dimension to the hydrogen bonding network in CuF_2_(H_2_O)_2_(3-chloropyridine) above 0.8 GPa (Fig. 1 (b)). Once established, this supplemental linkage, combined with improved in-plane superexchange (due to shorter distances between F centers and 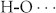), facilitates the pressure-induced antiferromagnetic to ferromagnetic crossover. The newly formed 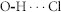 hydrogen bond decreases the angle of the 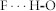 exchange pathway, making it even further away from the ideal 180° angle to support ferromagnetism. This means that the new hydrogen bond pathway must be the driving mechanism of the magnetic crossover. Since the 0.8 GPa magnetic crossover is driven by these local lattice distortions, the transition should be considered magnetoelastic rather than purely magnetic^41^,^42^,^43^. Moreover, the crossover is an excellent illustration of how pressure-induced changes in bond lengths and angles modify the transfer integral *t* which in turn modifies the exchange interaction *J*^12^,^33^. In this case, the mechanism even changes the sign of *J*.

**Figure 4:**
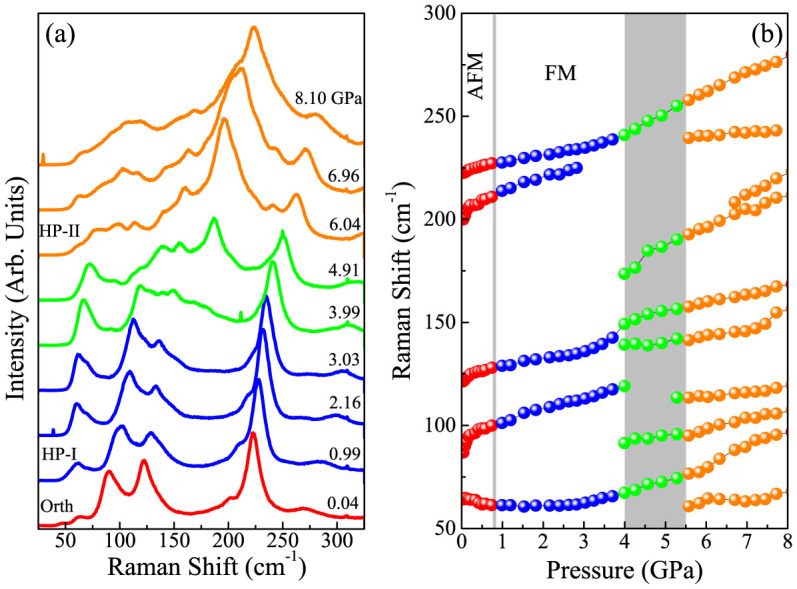
(a) Raman spectra as a function of pressure. The change in line color denotes a new phase (or coexistence of phases). (b) Raman shift versus pressure over the full pressure range investigated. The critical pressures are marked with grey vertical bands. The orthorhombic to high pressure phase I transition is at 0.8 GPa, and the broad transition with the coexistence of high pressure phases I and II is between 4 and 5.5 GPa. Here, Orth is *Pmna* orthorhombic (although at low temperature, the material is P2_1_/c monoclinic in this regime)^31^, HP-I is the first high pressure phase, HP-II is the second high pressure phase, AFM is antiferromagnetic, and FM is ferromagnetic. The magnetic phases are present at low temperature.

While the 0.8 GPa magnetic crossover in CuF_2_(H_2_O)_2_(3-chloropyridine) was previously identified (Schlueter, J. A. Unpublished work), there has been no investigation of structure at higher pressures. We extended our work up to 8 GPa and discovered an additional rather sluggish structural transition between 4 and 5.5 GPa. (Fig. 4) The low frequency Raman spectra are the most revealing in this regard. The appearance of five new modes, along with mode splitting at 120 cm^−1^ and the disappearance of the 85 cm^−1^ mode, signal the transition. The infrared-active modes show similar behavior in this pressure range (Supplemental Material). While we cannot precisely assign the new modes that appear, our dynamics calculations show that, in general, modes below 500 cm^−1^ are related to motion around the copper center, and those above 500 cm^−1^ are related to the 3-chloropyridine ring. Therefore, we conclude that this higher pressure distortion involves mostly the bipyrimidal copper environment, not the 3-chloropyridine ring. The increase in the number of vibrational modes through the transition indicates an overall reduction in symmetry around the copper center. It is clearly a lower symmetry subgroup of *Pnma* at 300 K and *P2*_1_/*c* below the structural phase transition temperature^31^. X-ray diffraction will be needed to identify the space group of the high pressure phases.

## Discussion

Having established the primary role of pressure-induced local lattice distortions in creating new hydrogen bonding pathways which in turn drive the antiferromagnetic to ferromagnetic crossover in CuF_2_(H_2_O)_2_(3-chloropyridine), we turn our attention toward prospects for control. One of the most important criteria in this regard is reversibility. As revealed by Fig. S4, the process is indeed reversible. Hydrogen bond networks form, diminish, and repeatedly reform under pressure. This implies that magnetism, which is determined by the dimensionality of the hydrogen bonding network that provides for superexchange between copper centers, is equally switchable. Whether this process can be demonstrated in thin film form and under lattice strain is an open question, but similar mechanisms involving coordinated motion of hydrogen bond networks that function as exchange pathways between magnetic centers are likely to play out in other quantum magnets. A secondary criteria is room temperature operation. The 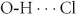 connections in CuF_2_(H_2_O)_2_(3-chloropyridine) are robust at 300 K. However, these connections only function as superexchange linkages at low temperature. We therefore anticipate that pressure- or strain-controllable exchange interactions^44^ can be realized only below the ordering temperature, although short range interactions might increase the operating temperature by a few degrees. Materials like V(TCNE)*_x_*·*y*(CH_2_Cl_2_) and (Et_4_N)_0.5_Mn_1.25_[V(CN)_5_]·2H_2_O may offer pressure- and/or strain-driven switchability at high temperatures^45^,^46^. Spin-crossover materials may be good candidates as well. Finally, this kind of cooperative functionality is not limited to piezomagnetism. Low power piezoelectric devices may also be possible if magnetoelectric coupling can be made strong enough^25^.

## Methods

CuF_2_(H_2_O)_2_(3-chloropyridine) was grown by slowly diffusing a vapor of 3-chloropyridine into an aqueous solution of CuF_2_(H_2_O)*_x_* as described previously^31^. Sample quality was confirmed by x-ray diffraction and magnetic susceptibility. A polycrystalline sample was loaded into diamond anvil cells either neat or with a pressure medium (vacuum grease for far and KBr for middle infrared) in order to apply quasi-hydrostatic pressure. The ruby fluorescence technique was used to measure the sample pressure inside the cell^47^. Raman measurements were performed with a 532 nm diode pumped solid state laser, with power below 1 mW to prevent sample degradation. Raman spectra were taken with a resolution of 0.5 cm^−1^, integrated between 60 and 120 seconds, and averaged three times. Infrared measurements were taken with a resolution of 1 cm^−1^ for all spectra. Due to the small sample size and 300 *μ*m diamond culets, the National Synchrotron Light Source at Brookhaven National Laboratory was used for its high brightness infrared light^48^. Standard peak fitting procedures were employed as appropriate. We also measured the variable temperature infrared response at ambient pressure and found no signatures of the orthorhombic to monoclinic transition between 200 and 100 K (Supplemental Material)^31^. Thus, we can connect our 300 K, high pressure measurements to the low temperature magnetic crossover. To understand the spectral results as well as the magnetic properties of CuF_2_(H_2_O)_2_(3-chloropyridine), we carried out multi-scale calculations in which both the molecular unit was modeled using molecular orbital theory and the magnetic properties under pressure were calculated via super cell techniques and band structure methods. Using density functional theory with the generalized gradient approximation, we calculated lattice dynamics of a single unit of CuF_2_(H_2_O)_2_(3-chloropyridine) as well as an isolated 3-chloropyridine ring and water molecule to assign the vibrational modes. The relative enthalpy of the antiferromagnetic and ferromagnetic states was calculated at various pressures using spin-polarized density functional theory. See Supplemental Materials for additional details.

## Author Contributions

J.L.M. conceived the project, developed the plan, and gathered the team. J.A.S. synthesized the material. K.R.O., T.V.B., J.B.W., C.M., Z.L. and J.L.M. performed the spectroscopic measurements. K.R.O. and J.B.W. analyzed the findings and discussed the data with J.L.M., Z.L., T.V.B., S.G., Q.W. and P.J. The theoretical calculations were performed by S.G., Q.W. and P.J. The paper was written by K.R.O., J.L.M. and P.J. with input from all coauthors.

## Supplementary Material

Supplementary Information Supplemental Material
