# Supplementary material for: Pressure-Induced Magnetic Crossover Driven by Hydrogen Bonding in CuF2(H2O)2(3-chloropyridine)
Source: Sci Rep. 2014 Aug 13;4:6054. doi: 10.1038/srep06054 (PMC4131223; doi:10.1038/srep06054)
Supplement: Supplementary Information — Supplemental Material [file srep06054-s1.pdf]

Supplementary Material for "Pressure-Induced Magnetic Crossover Driven by Hydrogen Bonding in $CuF_2(H_2O)_2$(3-chloropyridine)"

Kenneth R. O'Neal,[1] Tatiana V. Brinzari,[1] Joshua B. Wright,[1] Chunli Ma,[2,3] Santanab Giri,[4] John A. Schlueter,[5,6] Q. Wang,[4] Puru Jena,[4] Zhenxian Liu,[7] and Janice L. Musfeldt*[1]

[1]*Department of Chemistry, University of Tennessee, Knoxville, Tennessee 37996 USA*

[2]*Geophysical Laboratory, Carnegie Institution of Washington, Washington D.C. 20015, USA*

[3]*State Key Laboratory of Superhard Materials, Jilin University, Changchun 130012 China*

[4]*Physics Department, Virginia Commonwealth University, Richmond, Virginia 23284 USA*

[5]*Materials Science Division, Argonne National Laboratory, Lemont, Illinois 60439 USA*

[6]*Division of Materials Research, National Science Foundation, Arlington, Virginia 22230 USA*

[7]*Geophysical Laboratory, Carnegie Institution of Washington, Washington D.C. 20015 USA*

(Dated: July 17, 2014)

I. Room Temperature Vibrational Properties

In order to investigate the 0.8 GPa antiferromagnetic to ferromagnetic crossover in $CuF_2(H_2O)_2$(3-chloropyridine), we carried out high pressure spectroscopic measurements. The infrared and Raman spectra at ambient conditions (Fig. S1) display a large number of vibrational modes. Our lattice dynamics calculations allow us to assign the peaks below 500 cm^{-1} as displacements involving the bipyrimidal copper environment, for example the F-Cu-F symmetric and asymmetric stretches. These features are highly collective. Modes above this threshold originate from the 3-chloropyridine ring, or are well-known organic functional group vibrations such as the O-H (3200 cm^{-1}) stretch). Assignment of these modes (Tables S1 and S2) enables us to understand the role of the lattice in the pressure-driven magnetic crossover.

II. Variable Temperature Vibrational Properties

Although $CuF_2(H_2O)_2$(3-chloropyridine) displays an orthorhombic (Pnma) to monoclinic ($P2_1/c$) structural transition between 200 and 100 K[S1], our variable temperature infrared

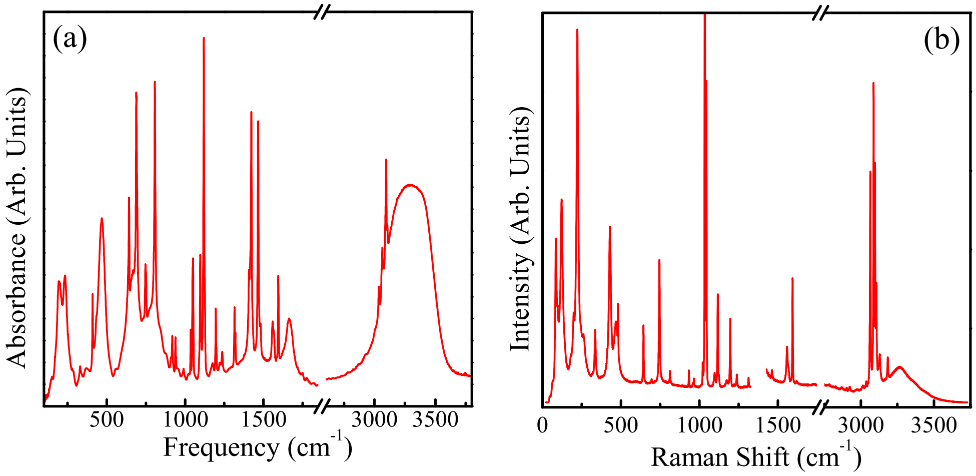

FIG. S1: Room temperature (a) infrared and (b) Raman spectra of $CuF_2(H_2O)_2$(3-chloropyridine).

measurements reveal no signature of symmetry changes down to 4.2 K (Fig. S2). In other words, although more vibrational modes are expected in the lower symmetry monoclinic phase as compared to the orthorhombic phase, the spectral patterns are equivalent. We therefore assume that pressure-induced changes in the room temperature orthorhombic phase are mirrored in the low temperature monoclinic phase. Short-range antiferromagnetic ordering is observed below 10 K, and long range ordering below 2.2 K in $CuF_2(H_2O)_2$(3-chloropyridine)[S1]. Our low temperature infrared spectra reveal no changes with the onset of short range magnetic ordering, indicating that antiferromagnetic ordering does not change the local structure. This allows us to use our room temperature high pressure measurements to understand the low temperature 0.8 GPa magnetic crossover.

III. Infrared Signatures of the Structural Transition Between 4 and 5.5 GPa

Beyond revealing the mechanism of the magnetic transition, we discovered an additional rather sluggish structural transition between 4 and 5.5 GPa. While there are many signatures of this higher pressure transition, the Raman spectra shown in the main text are the most revealing. The infrared active modes support this discovery as well (Fig. S3). As with the Raman active modes, the infrared features sensitive to the transition are mostly related to the pentacoordinate copper environment. The disappearance of five modes, the appearance of a new mode at 490 cm^{-1}, and the 190 cm^{-1} mode splitting all signal the structural transition. That they appear at low frequency indicates that there is a real lattice component to this

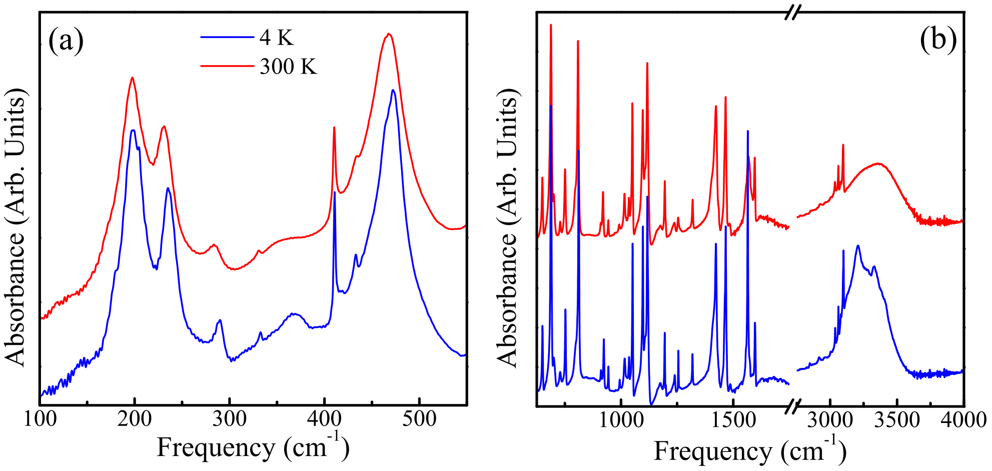

FIG. S2: 4 and 300 K spectra in the (a) far and (b) middle infrared. The spectra display no discernable changes of the orthorhombic to monoclinic transition between 200 and 100 K.

transition.

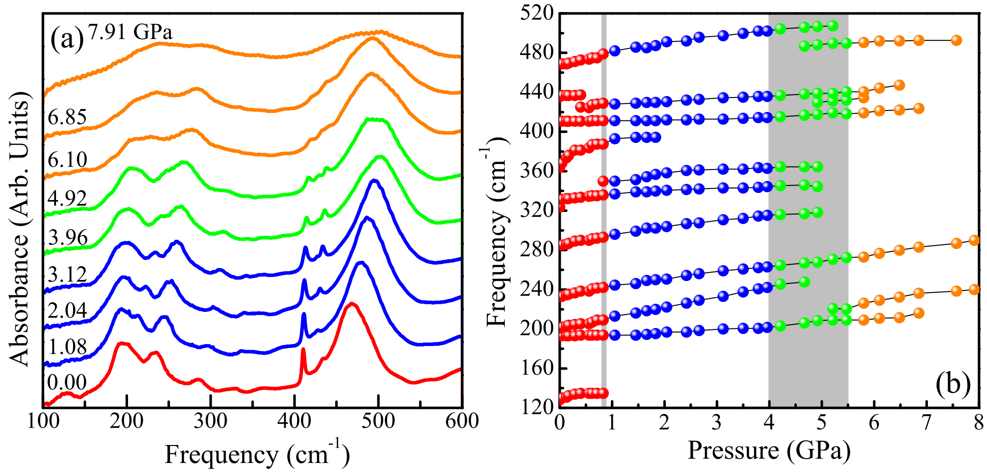

FIG. S3: (a) Infrared spectra at the indicated pressures. (b) Frequency vs. pressure for the infrared active modes. These modes related to the penta-coordinate copper environment also show sensitivity to the 4 to 5.5 GPa structural transition.

IV. Reversibility Considerations

We have shown in the main text that the 0.8 GPa includes a significant contribution from the lattice, making it a magnetoelastic transition. Moreover, a new structural crossover was discovered between 4 and 5.5 GPa. No other transitions were observed up to 11.5 GPa,

although many modes broaden significantly hinting at an onset of amorphization (Fig. S4). Upon the release of pressure, the system springs back into the low-pressure orthorhombic phase. Judging by the character of the spectrum, no damage was done. This reversibility allows for simple magnetostructural switching of $CuF_2(H_2O)_2$(3-chloropyridine) through the application and release of pressure, making it a candidate material for piezomagnetic applications.

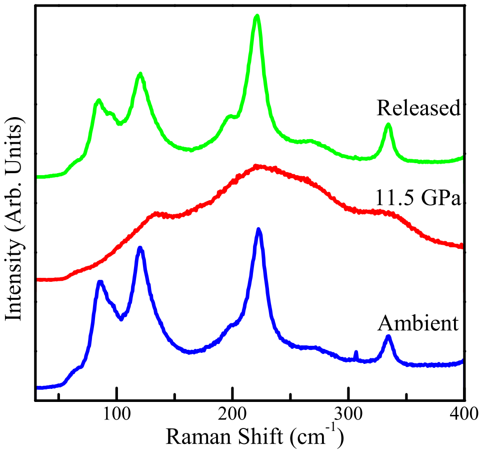

FIG. S4: Raman spectra at ambient conditions (blue), 11.5 GPa (red), and after the release of pressure (green). The ambient and released pressure spectra are nearly identical, demonstrating the lack of hysteresis.

V. Calculation Details

To understand the spectral results as well as the magnetic properties of the molecular crystal, we employed a multi-scale approach where both the molecular unit as a building block was modeled using molecular orbital theory and the magnetic properties under pressure were calculated using super cell techniques and band structure methods. We realize that many of the vibrational modes in the crystal are dominated by the inter-atomic vibrations of the molecular building block as these are linked in the crystal through hydrogen bonds.

In order to learn about the molecular vibrational modes we calculated the frequencies of a single unit of $CuF_2(H_2O)_2$(3-chloropyridine) taken from the crystal geometry. The calculations were based on density functional theory with generalized gradient approximation for exchange and correlation potential. Since $CuF_2(H_2O)_2$(3-chloropyridine) is bonded through

a hydrogen bonding network, we have used Grimme's functional[S2] including dispersion correction with the B97D[S2] functional and SDD[S3] basis as implemented in Gaussian09[S4]. The infrared and Raman frequencies were calculated without optimizing the geometry in the gas phase. Understandably, this led to a number of imaginary frequencies most of which come from 3-chloropyridine and H_2O. To circumvent this problem we have calculated the infrared and Raman spectra for individual 3-chloropyridine and H_2O units at the same level of theory. Figure S5 summarizes our results, and mode assignments are given in Tables S1 and S2.

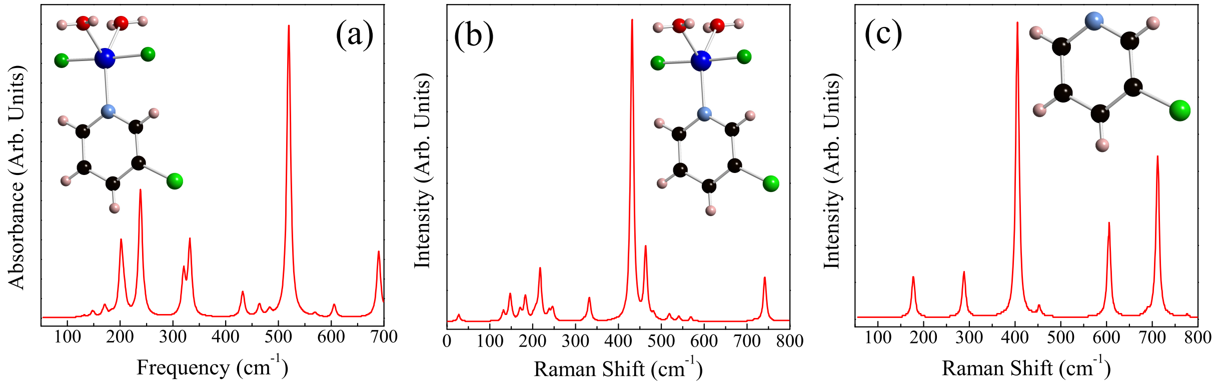

FIG. S5: Calculated (a) infrared and (b) Raman spectra of $CuF_2(H_2O)_2$(3-chloropyridine). (c) Calculated Raman spectrum of isolated 3-chloropyridine molecule. Insets show the molecule for which the spectra were calculated.

To understand the antiferromagnetic to ferromagnetic phase transition under pressure we have used spin-polarized density functional theory. The Vienna Ab initio Simulation Package[S5] based on Projector augmented wave method[S6] was used for geometry optimization. Wave functions were expanded in plane waves up to a kinetic energy cutoff of 500 eV. The electron exchange-correlation functional prescribed by Perdew-Burke-Ernzerhof[S7] within the generalized gradient approximation is used except as otherwise stated. Full ionic and supercell relaxation are performed at a given external pressure implementing the conjugate gradient algorithm. Convergence criteria for total energy and ionic force components were set at 10^{-4} eV/cell and 10^{-3} eV/Å, respectively. Since the crystal is bound by a network of hydrogen bonds, we used the modified density functional theory method of Grimme[S2] to properly treat the long range dispersive interactions. The Brillouin Zone was represented by means of 3x3x1 Monkhorst and Pack[S8] k points grid. Calculations using a denser grid

showed no qualitative improvement in the calculated results.

Because $CuF_2(H_2O)_2$(3-chloropyridine) magnetically orders below 2.1 K, we chose the low-temperature monoclinic phase to simulate the phase transition. We used enthalpy (H=U+pV) rather than merely total energy to assess the relative stability of different magnetic configurations as a function of pressure. Based on the experimental observations, both the ferromagnetic and antiferromagnetic configurations were relaxed at 0, 0.6, 0.8, 1, 2, 2.5, and 3 GPa pressure. The changes in relative enthalpy and volume with increasing external pressure were calculated. The relative enthalpy was found to be positive around 0.8 GPa implying an antiferromagnetic to ferromagnetic crossover.

TABLE S1: Assignment of the infrared active vibrational modes for $CuF_2(H_2O)_2$(3-chloropyridine) at ambient conditions. Values of $d\omega/dP$ are calculated only up to 0.8 GPa due to the phase transition and averaged for the C-H stretches.

experimental frequency (cm^{-1})	calculated frequency (cm^{-1})	calculated $d\omega/dP$ (cm^{-1}/GPa)	mode displacement pattern
128	148	7.1	pyridine ring rotation about C-Cl bond, F-Cu-F symmetric bend, O-Cu-O asymmetric bend
193	171	1.1	O-Cu-O torsion, F-Cu-F bend
201	202	9.4	Cu-N stretch, out of phase pyridine ring bend with C-Cl wag
233	238	11	in phase F-Cu-F bend
285	320	9.8	Cu-O asymmetric stretch
324	330	9.5	Cu-O symmetric stretch, out of phase symmetric Cu-F stretch
410	431	0.9	Cu-O and Cu-F in phase stretches
436	463	3.1	out of plane pyridine ring buckling with C-Cl stretch
469	483	11	pyridine ring distortion
580	520	3.95	F-Cu-F asymmetric stretch
643	606	4.0	ring buckling
690	689	1.3	in plane ring bend
806	826	1.2	ring breathing, in plane, in phase C-Cl stretch
1048	1048	2.0	in plane ring bend
1112	1104	2.0	ring breathing, out of phase C-Cl stretch
1558	1576	1.9	C=C and C-N in plane, out of phase stretch, C-Cl stretch
1595	1641	3.8	C=C and C-N in plane, out of phase stretch
1664	1586	3.9	H-O-H bend
3035-3105	3116-3155	7.0	C-H stretches
3197	3725	-28	O-H stretch
3394	3851	-31	O-H stretch

TABLE S2: Raman active vibrational mode assignments for $CuF_2(H_2O)_2$(3-chloropyridine) at ambient conditions. Values of dω/dP are calculated only up to 0.8 GPa due to the phase transition and averaged for the C-H stretches.

experimental frequency (cm^{-1})	calculated frequency (cm^{-1})	calculated dω/dP (cm^{-1}/GPa)	mode displacement pattern
64	28	-5.2	H_2O-Cu libration, F-Cu-F out of phase bend, C-Cl out of phase wag
87	132	16	C-Cl wag against molecule
122	148	8.7	pyridine rocking, F-Cu-F bend, O-Cu-O rocking, H-O-H libration
200	171	14	F-Cu-F and O-Cu-O bends, C-N stretch, C-Cl wag
222	183	6.7	out of phase F-Cu-F rotation, O-Cu-O bend
266	217	18	out of phase, out of plane pyridine ring wag
336	332	5.8	O-Cu-O symmetric stretch, in phase F-Cu-F bend
430	432	2.2	in phase F-Cu-F and O-Cu-O symmetric stretch
469	463	15	C-Cl stretch, pyridine ring motion, Cu-N wag
644	606	2.7	in plane ring bend, C-Cl wag
746	742	3.3	symmetric in plane pyridine ring bend, C-Cl stretch
1559	1575	2.8	in plane, in phase C=C and C-N stretch, C-Cl wag
1594	1642	4.9	in plane, in phase C=C, C-C, and C-N stretch
3060-3125	3112-3152	6.8	C-H stretch
3266		-19	O-H stretch

[S1] Lapidus, S. H. *et al.* Antiferromagnetic ordering through a hydrogen-bonded network in the molecular solid $CuF_2(H_2O)_2$(3-chloropyridine). *Chem. Comm.* **2013**, *49*, 499-501.

[S2] Grimme, S. Semiempirical GGA-Type Density Functional Constructed with a Long-Range Dispersion Correction. *J. Comput. Chem.* **2006** *27* 1787-1799.

[S3] Dunning, T. H. Jr. & Hay, P. J. *Modern Theoretical Chemistry*; Ed. Schaefer III, H. F. Vol. 3 (Plenum, New York, 1976) 1-28.

[S4] Frisch M. J. *et al.* Gaussian 09, Revision C.01, Gaussian, Inc., Wallingford CT, 2010.

[S5] Kresse, G. & Furthmller, J. Efficient Iterative Schemes for Ab Initio Total-Energy Calculations Using a Plane-Wave Basis Set. *Phys. Rev. B* **1996** 54 11169-11186.

[S6] Blöchl, P. E. Projector Augmented-Wave Method. *Phys. Rev. B* **1994** 50 17953-17979.

[S7] Perdew, J. P., Burke, K., & Ernzerhof, M. Generalized Gradient Approximation Made Simple. *Phys. Rev. Lett.* **1996** 77 3865-3868.

[S8] Monkhorst, H. J. & Pack, J. D. Special Points for Brillouin-Zone Integrations. *Phys. Rev. B* **1976** 13 5188-5192.
